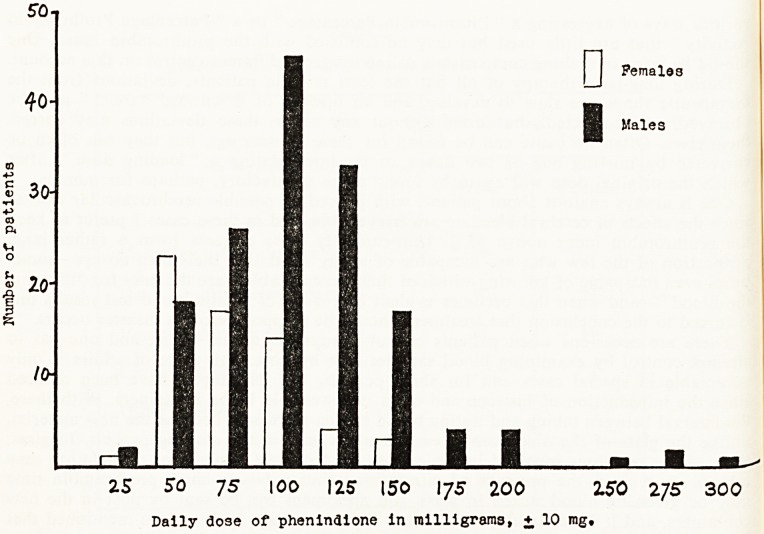# Experiences in the Control of Long Term Anticoagulent Treatment

**Published:** 1966-10

**Authors:** A. B. Raper

**Affiliations:** Pathology Department, United Bristol Hospitals


### EXPERIENCES IN THE CONTROL OF LONG-TERM ANTICOAGULANT TREATMENT

BY

A. B. RAPER

(*Pathology Department, United Bristol Hospitals*)

Long-term anticoagulant treatment has now been in use for 22 years, and has passed through the usual phases of over-enthusiasm, partial rejection, changes in emphasis, and adaptation to new therapeutic situations; but it is still with us. With its value this article is not concerned. I intend only to describe the problems that arise during the management of a group of patients for whom anticoagulants had been advised after investigation by experienced physicians and surgeons.

### THE CLINIC

All patients attended an out-patient clinic at the Bristol Royal Infirmary, usually after preliminary stabilisation had been achieved during a stay in hospital. Nearly all were ambulant, and many had returned to work, or did so while attending the clinic. The clinic was started to meet the needs of two of the interested parties. For the patients it offered the security of regular blood tests, a negligible waiting period, and appointments during the lunch hour, so that work was subject to the least interruption. For the laboratory staff it meant that tests could be conducted in a large batch (with a single normal control) once a week, and that every specimen could be relied upon as correctly taken, and could be tested without delay—all measures that added to confidence in the results.

A routine was strictly adhered to. Patients were seen from 11.30 a.m. to 1 p.m. Tests were performed between 2 and 3 p.m. If the result indicated a need for a change in anticoagulant dosage, a letter in standard form was posted that evening, giving clear instructions. Thus if no letter was received next day, the patient knew that all was well and that he should continue to take the same dose. Unvarying fulfilment of these rules was intended to give the patient confidence that he was receiving regular personal attention. On first attendance, every patient was told how the clinic was run, the nature and aims of treatment were explained, and he was told what effects he *might* observe in himself as the result of mild over-dose.

### THE PATIENTS

From January 1960 to June 1966, 238 patients attended for long-term therapy, 65 females and 173 males, the females for an average of 15 months each, and the males for an average of 20.5 months. The reasons for which anticoagulant therapy had been recommended were of course different for the two sexes (Table I), and they also reflect changes in medical opinion over the 6½ years of this review. In 1960-1963, males joining the clinic outnumbered females by 4.12 to 1, and they mostly suffered from coronary artery disease; in 1964-1966, as a result of the expansion of cardiac surgery and of the doubts cast on the value of anticoagulants as an insurance against a second or third cardiac infarct, the preponderance of new male patients was reduced to 1.24 to 1. In these latter years, valvular heart disease was the occasion for treatment in 24 of the 33 new female, and 7 of the 41 new male patients. Ten out of 65 females, but only 10 out of 173 males were under the age of 40 on first attendance, although the range of ages was about the same for the sexes, 26 to 80 for females and 24 to 72 for males.

TABLE I

Reasons for long-term anticoagulant treatment.

| Diagnosis | Number of patients | |
|---|---|---|
| | Male | Female |
| Coronary artery disease ... ... | 116 | 3 |
| Valvular disease of heart ... | 10 | 41 |
| Venous thrombosis ... ... | 21 | 16 |
| Carotid stenosis ... ... ... | 10 | 1 |
| Other diseases ... ... ... | 16 | 4 |
| Total | 173 | 65 |

Fig. 1.—Distribution of daily dosage of phenindione required for stabilisation, in males and females.

### LABORATORY TESTS AND CONTROL

The indispensable test is Quick's one-stage " prothrombin time " performed upon a correctly taken specimen with reagents of constant potency. When laboratories prepare their own reagents, and indeed even when they use a central supply, the results are seldom the same in different laboratories. But in any one laboratory the results are quite constant enough to ensure reliable control. There are other tests; but against the undoubted theoretical advantages of the two tests devized by Owren and his school—the " prothrombin and proconvertin " test and the " thrombotest "—are to be set the inconvenience of the reagents for the former and the cost of the latter; as well as the fact that in most workers' hands the theoretical advantages give, at best, only a marginal advantage in practice. However, two tests are better than one, and since the introduction of Owren's thrombotest reagent we have used this as an additional test in all cases. As in other centres using both tests, we found that when a patient was maintained within the therapeutic but safe range originally recommended for the thrombotest (5-15%), the prothrombin index usually remained too high; that is, a considerably greater dose of the anticoagulant could be given before the prothrombin index was depressed to a level known by experience still to be safe. At the other end of the scale, low thrombotest levels occurred in both adequately and over-treated patients, and did not distinguish between the two groups. Both tests were taken into account, but to ensure that the patient received the maximal anticoagulation consistent with safety, most reliance was placed upon the Quick test.

There are several ways of reporting the Quick test, and they may cause some confusion. The least obscure quotes the patient's " prothrombin time " in seconds, and that of a healthy control. Secondly, the ratio between these times may be quoted, the control time being reduced to unity; ratios between 2 : 1 and 3.3 : 1 represent the therapeutic range. Thirdly, the " Prothrombin Index " may be quoted; this is the ratio as above, expressed as a reciprocal and then converted into a percentage, so that the range mentioned above would by this method appear as 50% to 30%. Finally there are various ways of expressing a " Prothrombin Percentage " or a " Percentage Prothrombin Activity " that are little used but may be confused with the prothrombin index. One should be wary in making comparisons of reports from different centres on this account.

During long-term therapy of all but the least reliable patients, deviations from the therapeutic range are slow to develop, and an upward or downward " trend " may be observed, and corrected; but even without any action these deviations may correct themselves. Often no cause can be found for these wanderings, but they can often be corrected by missing one or two doses, or by interpolating a " loading dose "; after which the original dose will again be found to be satisfactory, perhaps for months.

One is always anxious about patients with proved or possible cerebrovascular disease since the effects of cerebral bleeding are irreversible, and in these cases I prefer to keep the prothrombin index above 45%. Unfortunately these patients form a rather large proportion of the few who are incapable of safely regulating their own dosage—sometimes even incapable of knowing which of their several tablets are the ones for "thinning the blood "—and when this becomes evident by reason of erratic blood test results one is forced to the conclusion that treatment should be stopped before a disaster occurs.

There are occasions when patients cannot always get to the clinic, and one has to attempt control by examining blood samples sent by post. This state of affairs is only acceptable in special cases and for short periods, but its dangers have been reduced since the introduction of lustrene and other non-wettable blood containers. With these, the interval between taking and testing blood can be increased, because the new material, unlike the glass of the older containers, does not initiate the clotting process. In glass, some of the reactions involved in the earliest (or " contact ") phase of coagulation may take place even in the presence of citrate, and thus a falsely short prothrombin time may be given by blood stored in glass. But specimens can be sent by post in the new containers, and it is reasonably safe to test them next day. It should be mentioned that these containers cannot be relied upon to be completely water-tight, and two precautions are necessary: the first is to see that the citrate solution has not leaked away or evaporated, and the second is to stopper firmly and pack securely to prevent the escape of blood.

### DRUGS AND DOSAGE

There is everything to be said for restricting the number of drugs used to the smallest possible, and for being familiar with the effects of this small number. I have used phenindione in 216 cases and warfarin in 19, and other drugs only occasionally. Each drug was given in two doses a day except when the total dose needed was very small, and then only one dose was given. Since a dose of either drug exerts its maximum " prothrombin " effect a day or more after ingestion, and the rise and fall of this effect is slow, the control is fairly smooth; indeed when fine adjustments of dosage are being made it is useful to take account of the total weekly, rather than of the daily dose. It also follows that one cannot assess the result of a change in dosage until several days have elapsed, and in most cases a week is not too long an interval. On first coming to the clinic, therefore, a patient attends weekly until the adjustments that are usually necessary after leaving hospital have been made. As soon as stability has been achieved (usually in 3 weeks) the interval between attendances is increased, and for those patients who over the months have shown themselves to be stable and reliable six visits a year are quite enough.

The dosage of phenindione needed for a steady therapeutic effect was, on average, 75 mg. a day for women and 110 mg. a day for men, a difference presumably attributable to differences in body weight. Four male patients needed 250 mg. a day or more (one, 300 mg.), and they were not all heavy men. When one plots against each daily dose the number of patients found to need that dose, there is a hint (Fig. 1) that these four men belong to a population different from the rest; but they are too few to support a theory that the population contains two modalities in respect of phenindione metabolism or of sensitivity of the coagulation mechanism that responds to this drug.

Side effects of drug therapy were encountered; none in the case of patients taking warfarin, but several times in those taking phenindione. Urinary discolouration was noticed by the more observant patients. It was sometimes attributed by them to urinary bleeding, but if a glance at a freshly voided specimen showed a clear, mahogany-coloured urine, they could be re-assured. An orange-brown stain in the centre of the palm occurs in some patients; so far I have seen this only in men, and they all said that they ingested the tablet from the palm. Perhaps they contaminated the spot with saliva, for one can easily imitate the brown stain in this way, and also show that phenindione turns brown in alkaline solutions, including saliva. Presumably women take their tablets more daintily.

Skin rashes occurred in 4 patients taking phenindione, and disappeared on a change of drug. Diarrhœa developed in 3 patients after they had been taking phenindione for several months, and it ceased when the drug was changed; in one case an extensive gastro-intestinal investigation that was being pursued independently was arrested by this simple therapeutic change. Fortunately none of the severe side-effects such as anæmia, jaundice, or renal failure, was encountered.

### HAEMORRHAGIC INCIDENTS DURING TREATMENT: DEATHS

To quote figures for the hæmorrhagic incidents reported by patients at the clinic: there were 19 incidents of spontaneous bruising, 16 of hæmaturia, 7 of epistaxis, 3 of rectal bleeding, 2 of bleeding from the gums, 1 of menorrhagia, and 1 of hæmospermia. Doubtless many more incidents of bruising and hæmaturia occurred than were reported. With equal certainty one could say that 3 of the attacks of hæmaturia had other causes, and occurred while the laboratory tests were steady at a safe level. No life was endangered by bleeding, but one patient was acutely uncomfortable with uretic colic early in his treatment, and had to be admitted to hospital. Since nearly all the incidents were of minor degree, they needed only an adjustment of the dosage, sometimes only the omission of one or two single doses, with a check on the prothrombin time the next week. This is preferable to stopping the drug, because if the bleeding is severe enough for this, it cannot wait for the natural " run-down " but needs more rapid arrest with vitamin K. It should be noted, however, that the rather cheerful picture presented here is based on incidents during the relative stability of long-term therapy, and that more dramatic incidents are liable to occur during the initial phase of stabilisation.

Several patients required dental extractions, and two required ophthalmic surgery. Single dental extractions can be quite safely performed without interrupting the treatment in thoroughly stabilised patients, though I always advise the patient to be sure to let his dentist know that he is taking anticoagulants. When the patient or dental surgeon is apprehensive, two days respite from treatment may be given before the extraction. Aspirin should of course be avoided for dental or other pain.

One is always vigilant when a patient gives a history of peptic ulcer, and he is especially warned of the danger of allowing a bleeding area to develop, the need for moderation in diet, and the importance of reporting any dyspeptic symptoms.

Thromboses occurred in 3 patients. One man of 60 had a femoral embolus, one man of 62 died from a combination of a recent myocardial infarct and a subdural hæmatoma, and one man of 60 died from mesenteric thrombosis (he was under supervision for only 1 month, and was privately taking large doses of Tab. Codeine Co.).

Besides the above 2 deaths in patients attending the clinic there were 7 others, of which 3 were due to mitral stenosis; no post-mortem examinations were made, and the contribution of the therapy to this outcome is unknown, except that the level of anticoagulation could be presumed to be " safe " at the time of death in at least 2 of the patients.

### THE VALUE OF THE CLINIC

Some of the advantages of regular attendance at a clinic have been noted. There are several other considerations, perhaps more imponderable. After a dangerous thrombotic incident, and an anxious period in hospital, the patient (particularly if he is elderly) re-enters the world in an apprehensive state. He is at first bemused by his new treatment; explanations already given to him sink in but slowly, and he needs repeated reasurrance. He is given this in several ways. The regular blood test represents the protection of impersonal but incorruptible " science ". Repeated brief conversations about the treatment make this at last familiar. He gradually sees the fulfilment of the promise, given to him on first attendance, that regular therapy, regular ways of life, and the passage of a month or two, will transform his outlook. And this is confirmed by the conversations that he inevitably hold with " old-timers " while he is waiting.

Confidence having been established, a long-term policy of rehabilitation can be embarked upon. Regularity and moderation in habits can be inculcated; advice can be given on minor bodily functions, exercise, sleeping habits, the process of weight-reduction (a matter of gradually re-educating the appetite, not of " diets "), even TV-watching. The regular blood tests are necessary; but the regular visits that they entail are of potent therapeutic value, for they give an opportunity to help the patient to learn the most important lessons needed for his new way of life—how to adjust to, and live with, his often permanent disability. I strongly suspect that (except where cardiac surgery is being prepared for) life is prolonged and rendered more enjoyable as much by these lessons as by the anticoagulant drug. As far as really long-term anticoagulant therapy is concerned, would it be unduly provocative to suggest that an anticoagulant clinic could be run just as confidently without anticoagulants?

## Figures and Tables

**Figure f1:**
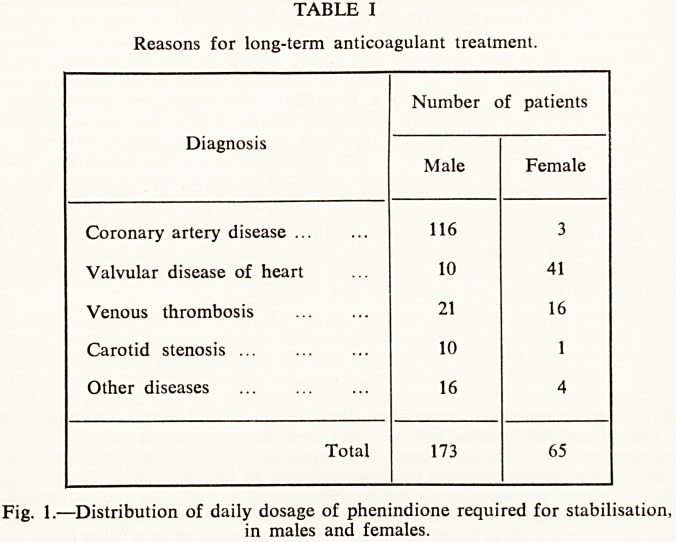


**Figure f2:**